# Identification of potential urine proteins and microRNA biomarkers for the diagnosis of pulmonary tuberculosis patients

**DOI:** 10.1038/s41426-018-0066-5

**Published:** 2018-04-11

**Authors:** Jieru Wang, Xiaojie Zhu, Xuekai Xiong, Pan Ge, Han Liu, Ningning Ren, Farhan Anwar Khan, Xia Zhou, Li Zhang, Xu Yuan, Xi Chen, Yingyu Chen, Changmin Hu, Ian D. Robertson, Huanchun Chen, Aizhen Guo

**Affiliations:** 1 The State Key Laboratory of Agricultural Microbiology, Wuhan, 430070 China; 2 College of Veterinary Medicine, Huazhong Agricultural University, Wuhan, 430070 China; 3 Tuberculosis Department, Wuhan Medical Treatment Center, Wuhan, 430023 China; 4 Hubei International Scientific and Technological Cooperation Base of Veterinary Epidemiology, Huazhong Agricultural University, Wuhan, 430070 China; 5 College of Veterinary Medicine, Murdoch University, Murdoch, 6160 Australia

## Abstract

This study identified urinary biomarkers for tuberculosis (TB) diagnosis. The urine proteomic profiles of 45 pulmonary tuberculosis patients prior to anti-TB treatment and 45 healthy controls were analyzed and compared using two-dimensional electrophoresis with matrix-assisted laser desorption/ionization time of flight mass spectrometry. Nineteen differentially expressed proteins were identified preliminarily, and western blotting and qRT-PCR were performed to confirm these changes at the translational and transcriptional levels, respectively, using samples from 122 additional pulmonary tuberculosis patients and 73 additional healthy controls. Two proteins, mannose-binding lectin 2 and a 35-kDa fragment of inter-α-trypsin inhibitor H4, exhibited the highest differential expression. We constructed a protein-microRNA interaction network that primarily involved complement and inflammatory responses. Eleven microRNAs from microRNA-target protein interactions were screened and validated using qRT-PCR with some of the above samples, including 97 pulmonary tuberculosis patients and 48 healthy controls. Only miR-625-3p exhibited significant differential expression (*p* < 0.05). miR-625-3p was increased to a greater extent in samples of smear-positive than smear-negative patients. miR-625-3p was predicted to target mannose-binding lectin 2 protein. A binary logistic regression model based on miR-625-3p, mannose-binding lectin 2, and inter-α-trypsin inhibitor H4 was further established. This three-biomarker combination exhibited better performance for tuberculosis diagnosis than individual biomarkers or any two-biomarker combination and generated a diagnostic sensitivity of 85.87% and a specificity of 87.50%. These novel urine biomarkers may significantly improve tuberculosis diagnosis.

## Introduction

Tuberculosis (TB) is a chronic infectious zoonotic disease caused by *Mycobacterium tuberculosis* (*Mtb*), and it is a significant worldwide threat to human health. The World Health Organization (WHO) reported that ~10.4 million people developed TB in 2016, and 1.7 million died, which makes TB the ninth leading cause of human death worldwide and the leading cause from a single-infectious agent^1^. TB is a great global burden to humans, and the WHO proposed an ambitious plan called “The END TB Strategy” (2016–2035) that targets a 95% reduction in TB death and a 90% reduction in TB incidence (<10 TB cases per 100,000) by 2035 based on 2015 data^2^. An accurate, rapid, and easy diagnosis is critical in the control of TB. TB diagnosis generally depends on clinical manifestations, tuberculin skin testing, smear microscopy, and bacterial culture isolation. However, the sensitivities and specificities for these methods are generally low. For example, isolation of *Mtb* from clinical samples is the gold standard for TB diagnosis. However, this test is a very low sensitivity and time-consuming methodology, and it requires a significant biosafety requirement for facilities^3^. Blood-based assays do more than just detect antibodies. The most commonly used clinical assay uses blood to detect cell-mediated immunity (e.g., interferon gamma production). However, the diagnostic sensitivity is suboptimal^4–6^. Some microRNA (miRNA) expression profiles of peripheral blood mononuclear cells (PBMCs) revealed differences between patients with active TB and healthy controls, which supports their potential application in TB diagnosis^7^. For example, miR-21, miR-142-3p and miR-223 were differentially expressed in active TB patients compared to healthy controls^8, 9^. The expression of miR-155 and miR-155* in PBMCs from active TB patients was upregulated after stimulation with the purified protein derivative^10^. ESAT6-dependent miR-155 inhibited the innate immune responses caused by *Mtb* infection and macrophage apoptosis induced by Bacillus Calmette–Guérin (BCG) infection^11–13^. The common procedure of these tests is blood collection, which is an invasive and painful sampling method. Therefore, exploration of novel diagnostic biomarkers with improved sensitivity, specificity, or easier sample collection using a minimally painful and non-invasive methodology must be developed.

Urine is an excreta that is not subject to homeostatic mechanisms. Therefore, fluctuations occur to a greater extent in urine than in blood, and changes in urine may reflect bodily status. Previous studies demonstrated that urine proteins could be used as potential diagnostic biomarkers in determining disease occurrence, development, and prognosis^14, 15^. For example, urine insulin-like growth factor-binding protein 1 (IGFBP-1) and secretory interleukin-1 receptor antagonist (sIL-1Ra) are markers for the early detection of lung cancer^16^, and urine α(1)-antitrypsin and epidermal growth factor (EGF) are markers for mouse and human atherosclerosis^17^. However, lipoarabinomannan (LAM) is the only biomarker for TB diagnosis confirmed in urine, and an assay kit for urine LAM for TB diagnosis is commercially available^18, 19^. The proteomics of urine based on all urine proteins of TB patients may reveal more potential biomarkers for the development of novel TB diagnosis methods.

The present study characterized potential urine biomarkers for pulmonary TB (PTB) diagnosis via comparisons of the urine proteomic profiles of PTB patients and healthy controls. Screening and confirmation studies demonstrated that two proteins, mannose-binding lectin 2 (MBL2) and a 35-kDa fragment of inter-α-trypsin inhibitor H4 (ITIH4-35k), and one miRNA (miR-625-3p) may be used in combination as urinary diagnostic biomarkers.

## Results

### Screening of different urine proteins between PTB patients and healthy controls

**Fig. 1:**
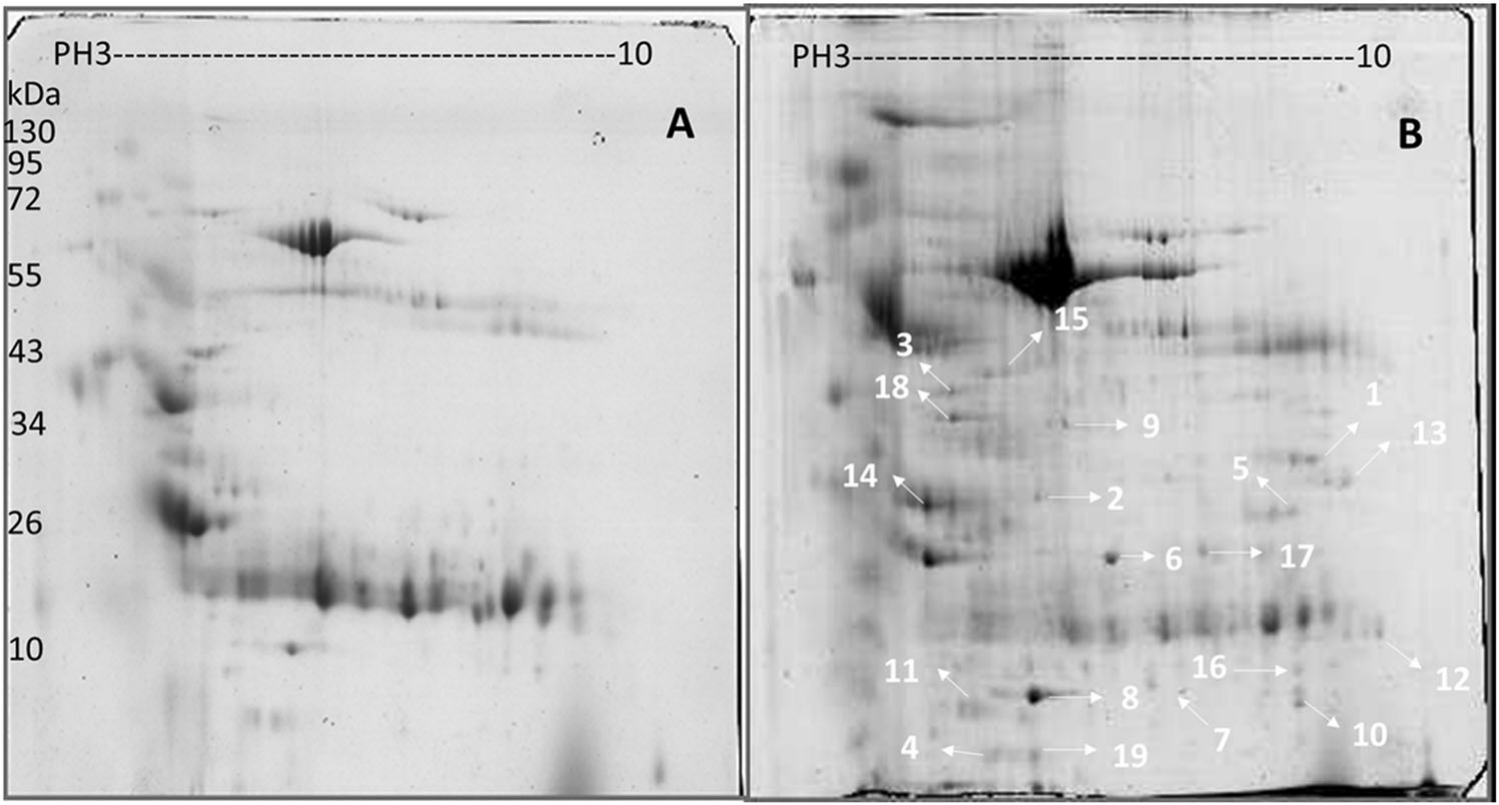
Representative 2-DE gel image obtained from pooled protein samples. Comparison of (**a**) healthy individuals and (**b**) pulmonary tuberculosis patient proteins in pH 3–10 separated on 2-DE gels. Isoelectric points are indicated at the top and molecular weight markers in kDa on the left. The data are representative of two separate experiments. A *p*-value <0.05 indicates statistical significance using the two-tailed *t*-test

Proteins in urine samples from PTB patients and healthy controls were separated using two-dimensional electrophoresis (2-DE) gel electrophoresis with a pH 3–10 gradient. Spot editing (spot splitting corrections and match editing) was performed sparingly only on selected areas of the gel. Statistical comparisons indicated that 19 spots were significantly different between PTB patients and healthy controls (encoded N1–N19), and these spots were identified using the default criteria of fold changes >1.50 and *p* < 0.05, which indicate a significant upregulation in PTB patients using an unpaired two-tailed *t*-test in the statistical tools built into the PD Quest software (Fig. 1). A total of 173 and 102 protein spots were identified from the urine samples of PTB patients and healthy controls, respectively, using 2-DE on the immobilized pH gradient (IPG) strip. Five protein spots representing MBL2 named N6, ITIH4-35k named N9, a transferrin variant named N5, microtubule-actin cross-linking factor 1 (MACF1) named N17, and transferrin protein named N10 were uniquely identified in PTB patients. The remaining 11 protein spots were upregulated in the PTB group compared to healthy controls. The top three proteins were mannan-binding lectin serine protease 2 (MASP2) named N8, an α-1-microglobulin/bikunin precursor (AMBP) named N3, and retinol-binding protein 4 (RBP4) named N4. Table 1 lists the protein identities, which were assessed using peptide mass finger printing (PMF) and peptide sequencing. These 19 proteins represented 29 different identities according to accession numbers. Several protein species were observed as multiple protein spots at slightly different pH values or molecular weights, or both, which suggests that some of these differences may be the result of post-translational modifications^20^.

**Table 1:** Characteristics of proteins identified in this study

Rank	Protein names	Accession no.	Protein score	Protein score C.I. %	Protein MW	Protein PI	Pep. Count
N1	Amylase,alpha2A; pancreatic variant (Fragment)	tr|Q53F26|Q53F26	501	100	58,368.3	6.6	17
N2	Albumin (fragment)	tr|F6KPG5|F6KPG	500	100	68,483.7	5.73	28
N2	Serum albumin	sp|P02768|ALBU_	189	100	71,317	6	5
N2	Cadherin-11OS = homo sapiens GN = CDH11 PE = 2	tr|H3BUU9|H3BUU	186	100	74,032.8	4.53	12
N3	Α-1-microglobulin/bikunin precursor. (AMBP)	gi|122801	423	100	39,886.3	5.95	7
N3	Thyroid receptor-interacting protein 11 isoform X3	gi|530404933	76	99	110,014	5	66
N4	Retinol-binding protein 4, plasma, isoform	gi|119570453	371	100	23,301	6	12
N4	Ataxin-7-like protein 2	gi|119588598	81	100	250,066	6	38
N4	Early endosome antigen 1, 162 kd	gi|93277115	67	92	78,785	9	19
N4	CD59 antigen, complement regulatory protein	gi|119617877	57	24	163,337	6	31
N5	Transferrin variant (fragment)	tr|Q53H26|Q53H2	334	100	79,311	7	8
N6	Lectin, mannose-binding 2, isoform	gi|119605421	318	100	26,812.3	5.12	8
N7	Basement membrane-specific heparansulfate proteoglycan core protein precursor variant	gi|62089288	314	100	249,966	7	17
N7	Chain a, crystal structure of protein hc from homo	gi|482677111	285	100	22,757.1	7.81	
N7	Prostaglandin-H2 d-isomerase	sp|P41222|PTGDS	130	100	21,243	8	10
N8	Mannan-binding lectin serine protease 2 isoform 1 preproprotein	gi|21264363	276	100	77,193	5	16
N9	35 kDa Inter-alpha-trypsin inhibitor H4	tr|B2RMS9|B2RM	225	100	103,526	7	
N10	Non-secretory ribonuclease precursor	tr|A0PJA6|A0PJA6	199	100	20,522	9	21
N10	TF protein (fragment)	gi|4506549	190	100	18,855	9	3
N11	Pro-epidermal growth factor EGF OS = homo sapiens	sp|P01133|EGF_H	176	99.668	17,983.4	7.42	5
N12	Alpha-1B-glycoprotein	sp|P04217|A1BG_	148	100	54,789.8	5.56	13
N13	Keratin, type II cytoskeletal 1	gi|119395750	148	100	66,170.1	8.15	20
N14	α-kinase anchor protein 9 isoform X2	gi|57015308	108	100	534,808.9	6.01	71
N14	Dynein, cytoplasmic 1, heavy chain 1	gi|530385399	75	98.765	458,794.5	4.96	63
N15	Gelsolin	tr|Q5T0H7|Q5T0H	101	100	20,827	5	13
N16	ALB protein	tr|Q8IUK7|Q8IUK7	89	99.984	46,441.9	5.77	14
N17	Microtubule-actin cross-linking factor 1	gi|578798796	75	99	878,150	5	
N18	NADH dehydrogenase [ubiquinone] complex I,	tr|C9JS27|C9JS27	62	91.289	29,973.4	5.63	9
N19	Acyl-coa-binding protein	sp|P07108|ACBP_	58	79.104	10,038	6.12	7

Protein score calculated by the Mascot search engine is the conformity between each protein with the MS peak list; confidence interval (C.I. %) for the protein score is statistical calculation of correlation between the acquired data and previous database under differing conditions using normal probability distribution mathematics by Mascot engine

*N* means number

### Bioinformatics analyses of proteomic data

**Fig. 2:**
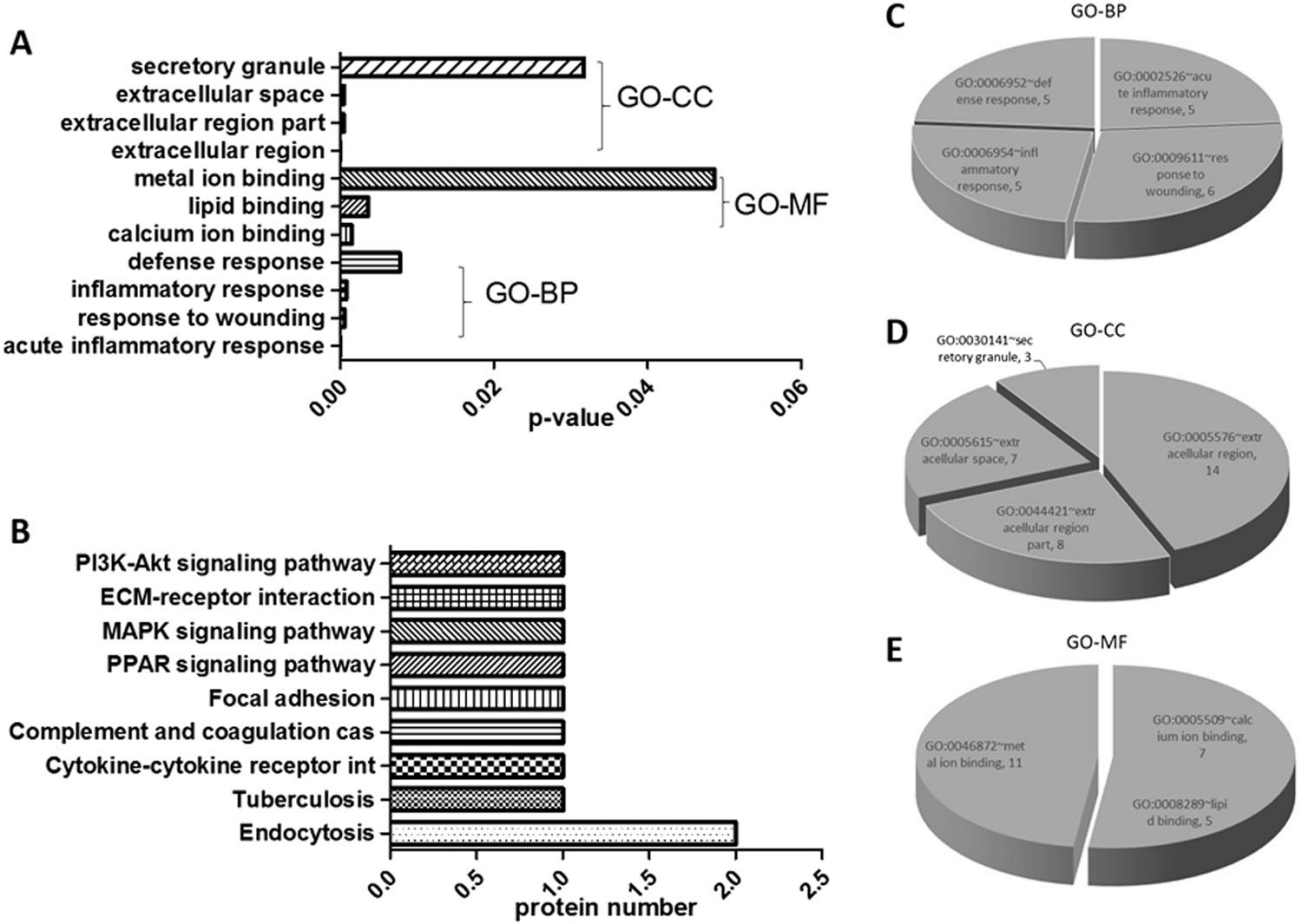
Data mining of the set of PTB urinary biomarker candidates. **a** Gene ontology (GO) enrichment analysis; **b** KEGG pathway mapping; **c** biological processes; **d** cellular component; and **e** molecular function. A *p*-value <0.05 indicates statistical significance using a binomial test

Gene ontology (GO) enrichment analyses were performed to classify human urinary proteins based on biological processes (BP), molecular function (MF), and cellular compartmentalization (CC). Enriched GO terms were obtained after the proteome of the healthy group was set as the background gene product, which included 11 terms containing four GO-BP terms, four GO-CC terms, and three GO-MF terms in the PTB group (Fig. 2). The following terms were obtained using David Pathway Analysis with a cutoff of *p* ≤ 0.05 and count >3 in a binomial test. The most significant GO-BP term for the PTB group was an acute inflammatory response (GO: 0002526) and an inflammatory response (GO: 0006954), both of which contained MBL2, TF, MASP2, and ITIH4-35k. The most significant GO-CC term for both groups was the extracellular region (GO: 0005576), which contained AMBP, MBL2, TF, RBP4, MASP2, ITIH4-35k, HSPG2, EGF, and AMY2A. The most significant GO-MF term for both groups was calcium ion binding (GO: 0005509), which contained MBL2, MACF1, MASP2, EGF, AMY2A, and CDH11.

We performed the following procedures to reduce the list of potential biomarkers to a smaller panel of proteins that would more likely predict PTB. Proteins that existed only in the PTB group and were significantly upregulated in the PTB group compared to healthy controls were clustered based on GO-BP, GO-MF, and GO-CC enrichment analyses. The following seven proteins were targeted for further evaluation of possible application in TB diagnosis: ITIH4-35k, MBL2, RBP4, MASP2, HSPG2, adherin-11, and AMBP in catalogues of the acute inflammatory response, extracellular region, and secretory granule, which were unique to individual terms in the PTB group.

### Verification of upregulated protein expression using urine from PTB patients

**Fig. 3:**
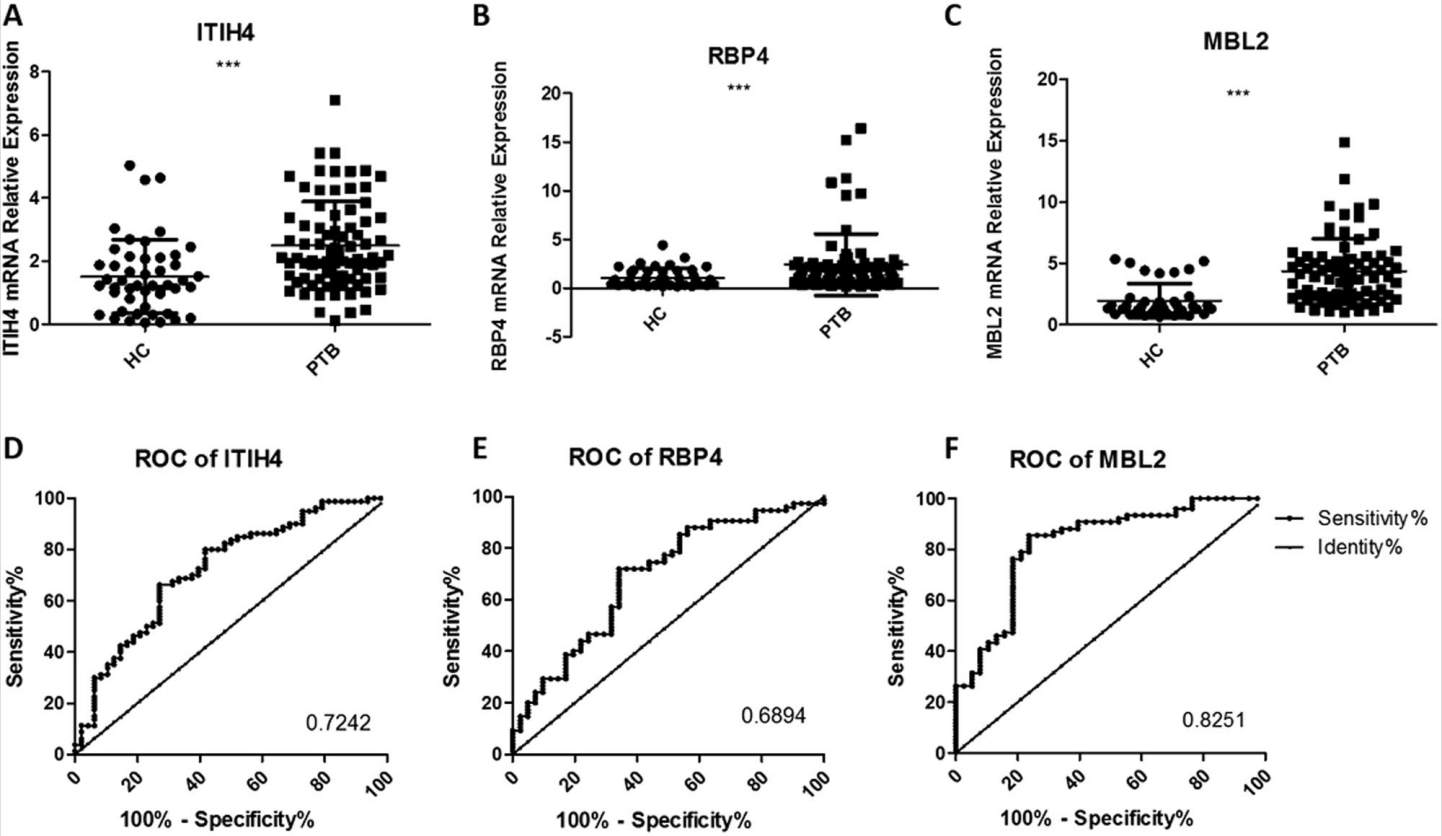
mRNA levels of three urine proteins in PTB patients and healthy controls. The relative contents of three identified urine proteins from tuberculosis patients and healthy controls determined using a qRT-PCR assay (**a**–**c**) and ROC curve analyses (**d**–**f**). The contents of the miRNAs were normalized to U6 and calculated using the 2^−ΔΔCt^ method. Each point represents the mean of triplicate samples. A *p*-value <0.05 indicates statistical significance with a non-parametric analysis using the two-tailed unpaired *t*-test. **p < 0.01, ***p < 0.001

We used SYBR green qRT-PCR with 97 urine samples in the PTB group and 48 samples in the control group to individually verify the seven critical candidate biomarkers, including MBL2, ITIH4-35k, RBP4, MASP2, HSPG2, cadherin-11, and AMBP, revealed in the bioinformatics analyses. ROC analyses based on the qRT-PCR assays were used to evaluate the sensitivities and specificities of the seven biomarkers. Urine MBL2 (accession number: P11226), ITIH4-35k (accession number: Q14624) and RBP4 (accession number: P02753) exhibited significantly higher levels of detection in PTB patients (*p* < 0.05) (Fig. 3). MBL2 was the best candidate biomarker, with a sensitivity of 76.32% (95% CI: 65.18–85.32%), a specificity of 78.95% (95% CI: 62.68–90.45%) and an AUC value of 0.8251 (Table S1). In contrast, the other four proteins exhibited no significant differences in transcription (*p* > 0.05).

**Fig. 4:**
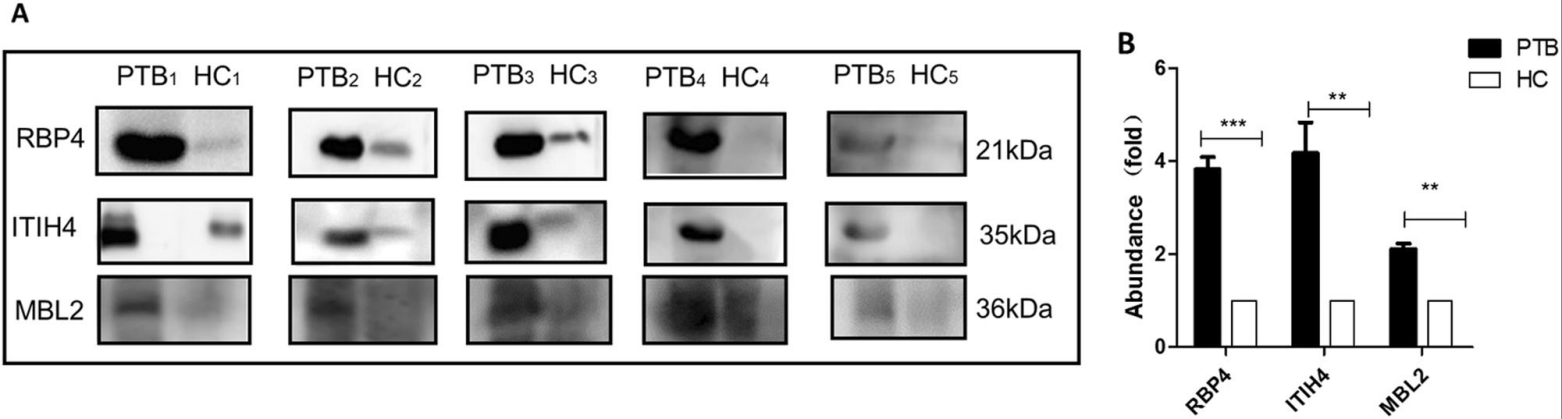
Comparison of candidate differential proteins using immunoblotting. Samples were analyzed using immunoblotting to facilitate comparisons of RBP4, ITIH4-35k, and MBL2 levels (**a**). Twenty-five pulmonary tuberculosis (PTB) patients and 25 healthy controls (HC) were randomly divided into five subgroups (PTB_1_ to PTB_5_; HC_1_ to HC_5_), each containing five individuals. The samples of each subgroup were pooled and detected in one lane, and each pair consisted of PTB patients and healthy control subgroups. **b** The densitometry quantification of the results of (**a**) using Image J software; *p*-values between the two groups with the two-tailed unpaired *t*-test. **p < 0.01, ***p < 0.001

The expression changes of these proteins in urine samples were confirmed using western blotting. Twenty-five urine samples were pooled into five subgroups for the PTB and control groups and tested. Protein bands on western blots were converted to grayscale images using ImageJ^®^ image processing program (NIH, Bethesda, MD, USA) and quantitatively compared. The expression levels of the same three proteins (ITIH4-35k, RBP4, and MBL2) were confirmed as the top three upregulated in the PTB group, with average changes of 4.18-, 3.82-, and 2.11-fold greater than those in the control group, respectively. There was a significant difference in expression levels between the PTB and control groups (two-tailed unpaired *t*-test, *p* < 0.01) (Fig. 4). MASP2 and AMBP were significantly upregulated (*p* < 0.05) with a <2-fold increase in expression levels, and there was no significant change in the expression level of the other two proteins (HSPG2 and cadherin-11, *p* > 0.05) (data not shown).

### Identification of miR-625-3p as a potential biomarker

**Fig. 5:**
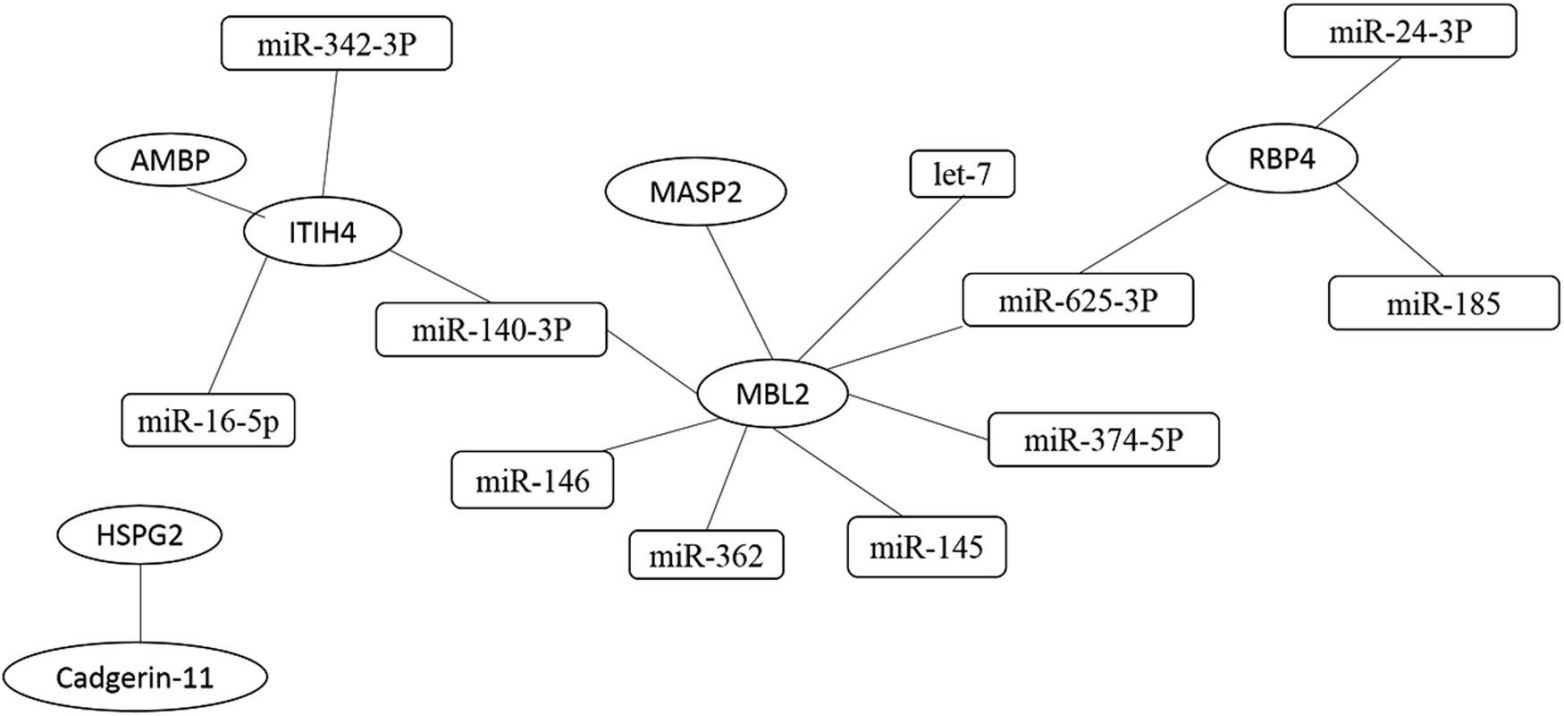
Integrative proteomics and transcriptomics analyses. Interactions between proteins are based on the reported literature. Interactions between proteins and miRNAs using four websites. The ellipse represents miRNA, and the rectangle represents proteins

**Fig. 6:**
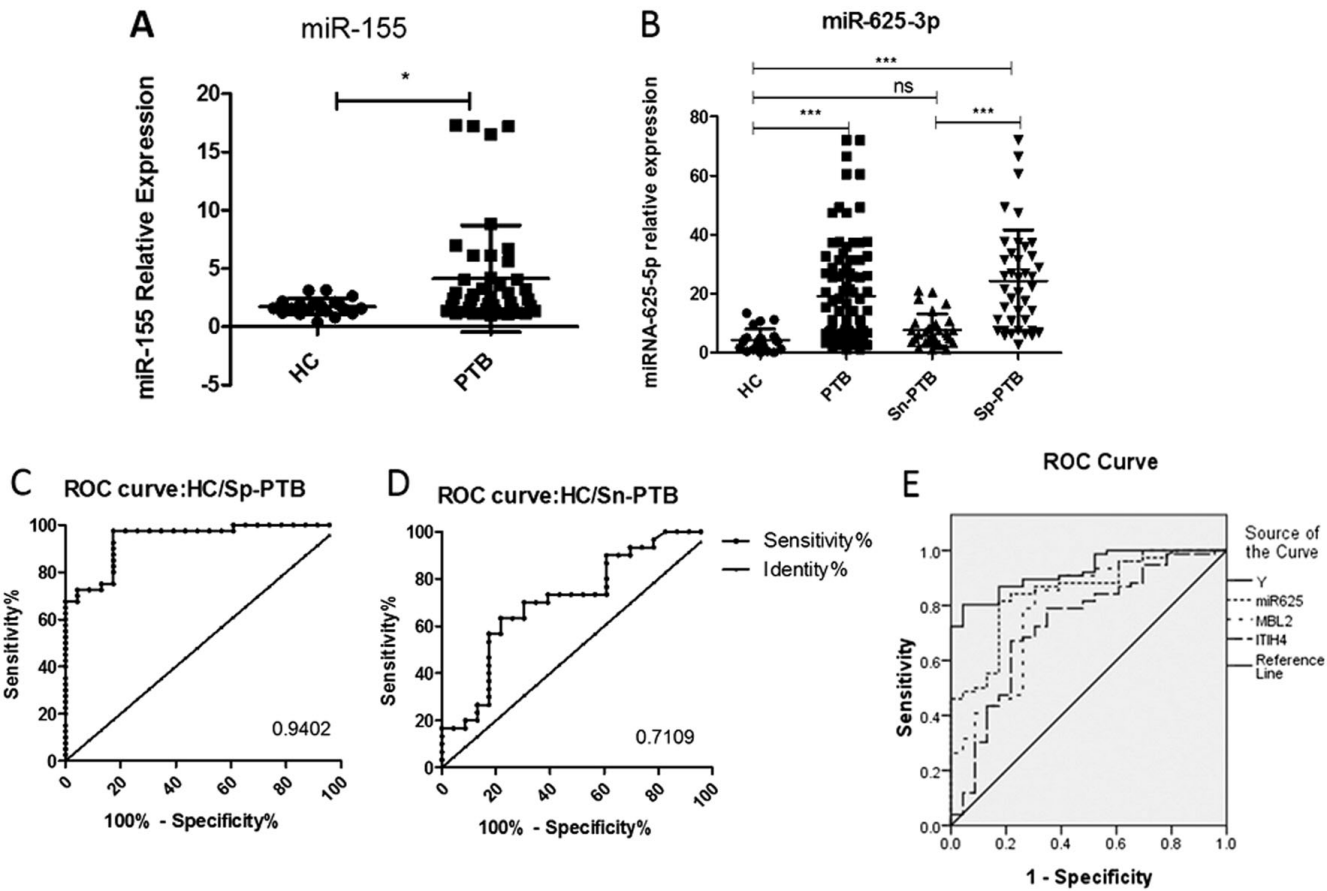
Urine levels of two miRNAs in Sn-PTB and Sp-PTB patients and healthy controls. **a** The expression of the positive control miR-155 in the PTB group and healthy controls; **b** the relative contents of miR-625-3p in Sn-PTB and Sp-PTB patients and healthy controls determined using qRT-PCR assay and ROC curve analysis between healthy controls compared with Sp-PTB patients (**c**); Sn-PTB patients compared with healthy controls (**d**); (**e**) the ROC analysis of Y (binary logistic regression model with miR-625-3p, ITIH4-35k, and MBL2), miR-625-3p, ITIH4-35k, and MBL2. The contents of the miRNAs were normalized to RNA U6. A *p*-value <0.05 indicates significance using the two-tailed unpaired *t*-test

The target miRNAs of the three differentially expressed proteins were first separately predicted using Targetscan, Miranda, MicroCosm, and PicTar, and a converged network was established based on this prediction. Eleven miRNAs were screened as a result: miR-374b-5p, miR-625-3p, miR-146, miR-140-3p, miR-145, let-7, and miR-362, which were related to MBL2; miR-16-5p, miR-140-3p, and miR-342-3p, which were related to ITIH4-35k; and miR-625-3p, miR-24-3p, and miR-185, which were involved in target gene RBP4 (Fig. 5). We further validated these miRNAs with SYBR green qRT-PCR using the urine samples from 97 PTB patients, including 46 Sp-PTB patients and 51 Sn-PTB patients, and 48 healthy controls and miR-155 as the positive control^10^. The positive control miR-155 exhibited significantly higher expression in the urine samples of PTB patients compared to healthy controls, which is consistent with previous research on serum miR-155 in PTB patients (Fig. 6a). The levels of the 11 urine miRNAs in urine samples were compared, and only the level of miR-625-3p in the PTB group was confirmed as being significantly higher than that in healthy controls (two-tailed unpaired *t*-test, *p* < 0.05) (Fig. 6b). Notably, miR-625-3p was predicted to target RBP4 and MBL2, which were significantly upregulated, as mentioned previously.

An ROC was created, and the accuracy of miR-625-3p was calculated. The results demonstrated that miR-625-3p as a diagnostic biomarker exhibited a sensitivity of 83.70% (95% CI: 74.54–90.58%), a specificity of 82.61% (95% CI: 61.22–95.05%) and an AUC value of 0.8599 in the differentiation of PTB patients from healthy controls. The upregulated miR-625-3p (*p* < 0.05) in PTB cases was subsequently validated in two independently arranged subsets of Sp-PTB/HC and Sn-PTB/HC. ROC analysis of miR-625-3p in the Sp-PTB/HC subset demonstrated that at a cutoff value of 0.9402, a sensitivity of 97.50% (95% CI: 86.84–99.94%) and a specificity of 82.61% (95% CI: 61.22–95.05%) were obtained (Table S2). The Sn-PTB/HC set exhibited a sensitivity of 70.00% (95% CI: 50. 60–85.27%) and a specificity of 69.57% (95% CI: 47.08–86.79%) in the differentiation of Sn-PTB patients from healthy controls when the AUC value of 0.7109 was used (Table S2).

### Logistic regression analysis of confirmed biomarkers for combined diagnosis

The performance of various combinations of miR-625-3p, ITIH4-35K, MBL2, RBP4, and miR-155 (as the positive control) in PTB diagnosis was evaluated. The test results of these five biomarkers were set as covariates, and the diagnosis was set as the dependent variable. Backward conditional binary logistic regression analyses for the five biomarkers were performed^21^. RBP4 (*p* = 0.073) and miR-155 (*p* = 0.116) were removed based on *p*-values >0.05. Spearman correlation analysis determined that no correlation existed among miR-625-3p, ITIH4-35K, and MBL2 (*p* > 0.05) using SPSS software. Therefore, miR-625-3p, ITIH4-35k, and MBL2 were used to develop a regression model. The model was constructed and expressed as *Y* = 0.329miR625-3p+0.639MBL2+0.746ITIH4-4.502. ROC confirmed that the combination of miR-625-3p, ITIH4-35k, and MBL2 exhibited the best accuracy for the diagnosis of PTB (Table S3). The logistic regression model further demonstrated a good fit to the data with the likelihood ratio test generating a −2 log likelihood value of 54.686 and a Nagelkerke *R*^2^ value of 0.623, and the Hosmer and Lemeshow test indicated no significant difference between the observed and predicted data (*p* = 0.388) (Fig. 6e). The AUC of the predicted probability (PRE) using this logistic regression model based on this combination was 0.926 greater than that for each single biomarker in detection. Therefore, we obtained an overall diagnostic sensitivity of 85.87% (95% CI: 77.05–92.26%) and a specificity of 87.50% (95% CI: 74.75–95.27%) for PTB diagnosis.

## Discussion

An early, accurate, simple, and cheap diagnosis of TB is essential to improve TB treatments. To increase the sample accessibility and the sensitivity and specificity of diagnostic tests, novel biomarker identification is an important approach to pinpoint these issues. Proteomic analysis is an efficient method to identify biomarker candidates. Therefore, the present study performed a urine proteomic assay to reveal potential novel urine biomarkers for PTB diagnosis. The urine proteomics profiles between PTB and healthy controls were compared, and seven differently expressed proteins were screened. qRT-PCR and western blot assays were used to confirm the previous results at the transcription and protein levels, respectively. The target miRNAs of confirmed proteins were predicted and validated to further improve the diagnosis. Logistic regression analysis demonstrated that the combination of three biomarkers, MBL2, ITIH4-35k, and miRNA-625-3p, performed best for the diagnosis of PTB.

MBL2 is a collagen-like serum protein that is a key component of innate immunity. MBL2 binds pathogens (*Mtb*) in its carbohydrate-recognition domains, and it mediates the lectin-dependent activation of the complement pathway^22, 23^. Plasma MBL2 levels from TB patients were higher than those from healthcare workers in Vietnam, which supports our findings^24^. However, the samples in this previous study were collected from smear-positive PTB patients with 0–7 months of treatment. Therefore, whether the MBL2 levels in urine were higher before or after treatment was not known. We provide the first evidence of significantly increased MBL2 levels in the urine of PTB patients prior to the initiation of anti-TB treatment compared to healthy controls in China. MBL2 is synthesized in the liver and circulates in the form of oligomers complexed with MBL2-associated serine proteases (MASPs)^25^. MASP2 is an enzyme of the innate immune system that is activated when one of these serine proteases recognizes microorganisms and subsequently cleaves complement factors C4 and C2 to initiate activation of the complement system^25^. MASP2 was upregulated in the urine of PTB patients, which further confirmed the findings of increased MBL2 levels.

ITIH4 is an acute-phase inflammatory response protein that belongs to a superfamily of protease inhibitors associated with a constant light chain of the AMBP type^26, 27^. Winden et al. reported ITIH4 detection up to 3 years prior to the diagnosis of some diseases, such as breast cancer and colorectal cancer, which supports its potential use as a diagnostic biomarker^28, 29^. AMBP is another acute-phase inflammatory response protein that was previously reported as being elevated in serum samples of cattle with subclinical *M. bovis* infection^30^. This evidence indirectly supports our findings regarding ITIH4-35k, although different types of samples were used; the former study used sera, while we used clinical urine samples.

The immune-related protein RBP4 is an adipokine that contributes to insulin resistance; RBP4 likely modulates pathophysiological processes during bacterial infection^26^. Two studies demonstrated that RBP4 levels were elevated in the serum of cattle infected with *M. bovis* and the whole-blood plasma of humans infected with *Mtb*^30, 31^. Young et al.^32^ reported a significantly increased level of RBP4 in the urine of active TB patients compared to other groups using SDS-PAGE and LC–MS/MS analyses, but without further confirmation. The present study further validated the changes at the translational and transcriptional levels in clinical urine samples. However, the sensitivity and specificity of this biomarker in PTB diagnosis were lower than those of the other biomarkers, and we excluded it from our final combination of three biomarkers. Overall, the present study provides preliminary evidence of the potential of MBL2 and ITIH4-35k as novel PTB diagnostic biomarkers in clinical urine samples.

Other investigators used urine miRNAs as diagnostic biomarkers for other diseases, such as atopic dermatitis and high-risk hepatitis C^33, 34^. Several studies reported that urine miRNAs were present in a highly stable, extracellular form, with Argonaute 2 (Ago2) complexes, which protect circulating miRNAs from RNases^35^, and are good candidates for use as biomarkers^36–38^. The amount of miRNAs in urine is lower than that in blood, but the extraction of miRNAs is simple and rapid using commercial reagents that require small amounts of miRNA (200 μL) for sensitive qRT-PCR detection. Therefore, we further investigated urine miRNA biomarkers. Recent studies reported the potential of some miRNAs as biomarkers in TB diagnosis and their roles in MTB-induced immunity and pathogenesis, but most of these studies used serum or whole-blood samples. The present study used urine samples to confirm that the serum TB-associated miR-155 was upregulated similarly in the urine of PTB patients, and we identified miRNA-625-3p as a good diagnostic urinary biomarker. A previous study reported that blood miRNA-625-3p discriminated malignant from benign lung tumors^39^. To our knowledge, the present report is the first study to demonstrate that urine miRNA-625-3p differentiated PTB from healthy controls.

To obtain the best performance using the validated biomarkers, we conducted logistic regression analysis. Then, ROC was used to evaluate the prediction from the logistic regression model. The combination of MBL2, ITIH4-35k, and miRNA-625-3p exhibited the highest sensitivity and specificity for PTB diagnosis.

Only one urine biomarker, LAM, is currently used for TB diagnosis. The combination of the LAM assay with sputum microscopy suggests an increase in the sensitivity for TB diagnosis but a decrease in specificity^40^. The present study demonstrated that three novel urine biomarkers would provide a better diagnostic method for PTB, and this finding would significantly improve PTB diagnosis.

However, the present study had several limitations. First, urinary samples for biomarker investigation were collected prior to the initiation of anti-TB treatment. Whether these biomarkers are altered during treatment and during relapse stages require further evaluation. Second, the sample number used for screening and confirmation was limited, and the results require further confirmation using more clinical samples. Third, the urine samples were frequently not stable during storage. Therefore, optimal methods to stabilize urine proteins should be investigated.

## Materials and methods

### Ethics statement

Physicians at Wuhan Medical Treatment Centre collected human urine samples from healthy donors and pulmonary tuberculosis patients in accordance with the Measures for the Ethical Review of Biomedical Research Involving Human Subjects issued by the National Health and Family Planning Commission of The People’s Republic of China. The Ethics Committee of Wuhan Medical Treatment Centre approved the study protocol (#2015001). Written informed consent was obtained from all participants involved in the study.

### Sample collection

Patients who were initially diagnosed with PTB and did not initiate anti-TB treatment were eligible for enrollment in the sample collection. Female participants in their menstrual cycle were excluded.

Urine samples were collected for various tests, and the maximum amount provided for this study was 50 mL/person. However, the three experimental stages, screening, confirmation, and verification, required ~75 mL/person. Therefore, the experiments at the three stages used independent urine samples and sample pools when necessary from TB patients and healthy controls. A total of 167 PTB patients and 118 healthy controls with a background of BCG vaccination were sampled during the entire study period. The sample numbers used in each stage of the experiments are detailed below.

We pooled the 50-mL urine samples from 45 PTB patients and 45 healthy controls to obtain urine samples of 2.25 L for each pool for the screening of potential urine biomarkers using 2-DE and matrix-assisted laser desorption/ionization time of flight mass spectrometry (MALDI-TOF-MS). The differential proteins were obtained via comparison of the proteomics profiles of the two groups.

Differential proteins were confirmed using western blot analysis and SYBR green quantitative reverse transcription polymerase chain reaction (SYBR green qRT-PCR). Urine samples from 25 patients with pulmonary TB and 25 healthy controls (HCs) were collected to confirm the western blot results. Urine samples from five individuals constituted a sub-pool, which were designated PTB_1_ to PTB_5_ and HC_1_ to HC_5_ for the patient and healthy control groups, respectively. Urine samples from 97 PTB patients and 48 healthy controls were individually tested to confirm the qRT-PCR results. A total of 46 of the 97 PTB patients were sputum smear positive (Sp-PTB), and 51 PTB patients were sputum smear negative (Sn-PTB). Table 2 lists the related information of the participants.

**Table 2:** Information of TB patients and healthy control

Characteristic	Active pulmonary PTB (*n* = 167)			Healthy control (*n* = 118)		
	2-DE screening	Validation tests		2-DE screening	Validation tests	
		WB	qRT-PCR		WB	qRT-PCR
*Total Number*	*45*	*25*	*97*	*45*	*25*	*48*
Age (y)	32 ± 9.2	40 ± 5.1	35 ± 6.3	29 ± 10.3	28 ± 10.4	32 ± 8.7
Sex (M/F)	30/15	16/9	52/45	28/17	14/11	26/22
Fluorescent sputum smear(±)	23/22	15/10	46/51	/	/	/
*Biochip tests*						
LAM(±)	22/23	19/6	89/8	/	/	/
16 kDa protein(±)	33/12	15/10	84/13	/	/	/
38 kDa protein(±)	34/11	19/6	90/7	/	/	/
Chest X-ray and CT-proven	36/9	23/2	93/4	—	—	—
PPD(±)	35/10	21/4	88/14	4/41	6/19	8/40
IGRAs(±)	29/16	23/2	92/5	1/44	3/22	4/44

Values expressed as means ± SD, number of patients, or number (percent)

*PTB* pulmonary tuberculosis, *WB* western blot, *2-DE* 2-dimensional electrophoresis, *LAM* lipoarabinomannan, *PPD* purified protein derivative of tuberculin, *IGRAs* IFN-γ release assays

PTB patients were diagnosed using the following tests:^41^ (1) demonstration of typical clinical signs, such as fever, coughing and productive sputum; (2) suggestive chest X-ray and computed tomography (CT) images; (3) three biochip tests of *Mtb* antibodies to specific antigens, including the 16-kDa protein, 38-kDa protein, and LAM (DeVID, Nanjing, China), with at least one positive test; (4) positive or negative fluorescence sputum AFB smear; and (5) a positive *Mtb* isolation in sputum culture. Cases were defined as PTB using the following criteria: satisfied criteria (1), (2), and (5) above and positive results on at least one of (3) and (4). All PTB patients were HIV-negative and had no other immunocompromised conditions, such as nephropathy or genitourinary disorders, which were determined using urinary sediment examinations and renal function tests. The healthy controls with a background of BCG vaccination were volunteers who were recruited randomly from individuals undergoing regular physical examinations. Healthy controls were defined using the following criteria: (1) no fever, coughing, or other clinical signs of PTB; (2) normal physical and radiography image results; and (3) no HIV infection, nephropathy, or genitourinary disorders. The IFN-γ in vitro release test (QuantiFERON-TB Gold, Neobioscience Technology, Beijing, China) and tuberculin skin test with purified protein derivation were performed, but the results were not considered in the PTB or healthy control definitions because all persons are compulsorily vaccinated with BCG in China.

### Urine collection and pretreatment

First midstream urine specimens from each individual (50 mL per urine sample) were collected in the morning using sterile tubes. Samples were equally combined to create sample pools, supplemented with a complete™ protease inhibitor tablet (Roche Diagnostics, Mannheim, Germany) to prevent proteolysis, and centrifuged at 4000×*g* for 20 min at 4 °C to remove cell debris and insoluble solids. The supernatant was filtered through a 0.22-µm filter (Millipore, Bedford, MA, USA) to remove contaminated bacteria and concentrated at 4 °C to ~1/40 of the initial volume using 3-kDa cutoff centrifuge tubes (Millipore). The resultant solutions were dialyzed at 4 °C for 48 h against 10 volumes of 18.2 MΩ cm^−1^ ultrapure water, which was changed eight times during this period to decrease the salt concentration^15, 16^. The concentrated urine specimens were stored at −80 °C for subsequent protein extraction.

### Urine protein precipitation

Urine proteins were further extracted as previously described^15^. Briefly, proteins were precipitated with 20% trichloroacetic acid (TCA) overnight at 4 °C and centrifuged (15,400×*g* for 20 min at 4 °C). The protein pellet was washed twice with cold acetone to remove TCA and re-suspended in rehydration buffer [8 M urea, 2 M thiourea, 0.5% CHAPS, 0.52% (v/v) ampholytes with pH range between 3 and 10, 60 mM DTT, and 40 mM Tris-HCl, pH 8.8] (Sigma-Aldrich, Shanghai, China). The protein concentration was determined using the 2-D Quant Kit (GE Healthcare, Shanghai, China). The final solubilized proteins were used immediately or stored at −80 °C.

### Urine protein separation using 2-DE

IPG strips in 2-DE were used as previously reported^42, 43^. Briefly, 17-cm IPG strips (pH 3–10) (Sigma-Aldrich) were rehydrated actively at 50 V, and urine protein samples (3 mg/strip) were placed in rehydration buffer with 0.002% (w/v) bromophenol blue for 16 h at 20 °C in a protean isoelectric focusing (IEF) cell (Bio-Rad, Hercules, CA, USA). IEF was performed under the following conditions: 150 V for 3 h, 300 V for 3 h, 1000 V (gradient) for 4 h, 10,000 V (gradient) for 5 h, and 10,000 V up to 60 kV-h. The strips were equilibrated in 10 mL of equilibration buffer (65 mM DTT in 6 M urea, 50 mM Tris-HCl, pH 8.8, 30% v/v glycerol, 2% w/v SDS, and 0.001% v/v bromophenol blue) for 15 min and transferred to 10 mL of equilibration buffer containing 135 mM iodoacetamide instead of dithiothreitol. The equilibrated IPG strips in 0.5% low-melting point agarose were placed on top of a 10% acrylamide separating gel (pH 8.8) and run at a constant voltage of 100 V at 20 °C using the Protean II xi multi-cell with a 2-DE conversion kit (Bio-Rad) until the dye front reached the end of the gel. Each experiment was independently performed twice. The stained gels were scanned using a GS-800 calibrated densitometer (Bio-Rad, Hercules, CA, USA), and the images were exported to PD Quest 8.0 software (Bio-Rad, Hercules, CA, USA) for image analyses.

### Identification of urine proteins using MALDI-TOF-MS

Different proteins were removed from the gels and further analyzed using MALDI-TOF-MS as previously described^43^. Collective PMF and MALDI-TOF-MS inquiries used the MASCOT search engine 2.2 (Matrix Science, Ltd., US) and the GPS Explorer Software, version 3.6 (Applied Biosystems, Foster City, CA, USA) from the NCBI database (Taxonomy: NCBI human134919). We selected the SWISSPROT *Homo sapien* database using the following default parameters: a peptide tolerance of 1.0 Da and a specificity of one missed cleavage; carbamidomethyl modification of cysteine; and acetylation of the NH_2_-terminal ends of lysine^43, 44^.

### Bioinformatics analyses of proteomic data

The MALDI-TOF-MS proteomic data were deposited in the Proteome X change Consortium (http://proteomecentral.proteomexchange.org) via the PRIDE^45^ partner repository with the dataset identifier PXD003479. The CC, MF, and BP were analyzed using the GO database (http://geneontology.org/). The Kyoto Encyclopedia of Genes and Genomes (KEGG) pathway mapping was performed using the KEGG Mapper (http://www.genome.jp/kegg/mapper.html).

### Prediction of PTB-associated miRNAs

Based on the MALDI-TOF-MS proteomic data, an interaction network of the differentially expressed proteins and related miRNAs was obtained using Cytoscape software, version 3.2.1 (http://www.cytoscape.org/)^46^. Four computational target prediction algorithms (http://www.targetscan.org, http://www.microrna.org, http://www.pictar.org, and http://www.ebi.ac.uk/enright-srv/microcosm/) were used to predict the binding sites in the corresponding genes targeted by differentially expressed miRNAs. The data predicted by these algorithms were combined, and the overlaps were calculated. The resultant differentially expressed miRNAs were further confirmed using qRT-PCR. Serum miR-155, which is associated with PTB, was chosen as the positive control to confirm the differential urine expression.

### Confirmation of mRNA and miRNA transcripts using SYBR green qRT-PCR

Total RNA was extracted from individual urine samples using the RNApure Total RNA Kit (Aislab Biotech, Beijing, China) for mRNA detection. Briefly, 1000 μL of QIAzol lysis reagent was added to 200 μL of urine in tubes, mixed, and incubated at 15–25 °C for 5 min. Chloroform (200 μL) was added, and the tubes were vortexed. The samples were centrifuged at 15,400×*g* for 15 min at 4 °C. The upper aqueous phase was transferred to a new tube, and a 1.5× volume of 100% ethanol (v/v) was added. The mixture was applied to a binding column. The column was washed twice, and the RNA sample was eluted from the column membrane into a collection tube using elution buffer. Urine miRNA was extracted from individual urine samples using the miRNeasy mini kit (Qiagen, Hilden, Germany) for the miRNA assay according to the manufacturer’s instructions.

Five microliters of eluted materials (total RNA or miRNA) was used as the template in a 25-μL reverse transcription reaction system using the All-in-One™ microRNA assay and Reverse Transcription Kit (Genecopoeia, Germantown, PA, USA). The reaction mixture was incubated in a 0.2-mL RNase-free strip tube for 60 min at 37 °C and 5 min at 85 °C and then held at 4 °C. A fixed volume of cDNA (6 μL), the All-in-One™ PCR mix and specific All-in-One™ Universal Adaptor microRNA assay primers were added. The reaction mixture was incubated in an Applied Biosystems 7500 Fast qPCR system (Applied Biosystems) at 95 °C for 10 min, followed by 40 cycles of 95 °C for 15 s and 60 °C for 1 min. All reactions were performed in triplicate with a blank control (distilled water) without cDNA. To compare the relative levels of urine miRNAs, RNA U6 was used as the internal reference for the miRNA and mRNA determinations^47^. The 2^−ΔΔCt^ method was used to calculate the relative levels of urine miRNAs. Table S4 lists the primers^23, 48, 49^.

### Confirmation of protein expression using western blot analyses

Western blot analyses were performed to confirm the differential expression of urine proteins. Briefly, 20 μg of urine proteins was separated using 10% (w/v) SDS-PAGE and transferred onto PVDF membranes. Membranes were blocked with 5% skim milk powder at room temperature for 3 h and incubated with mouse anti-RBP4 IgG (1:1000 dilution) (Proteintech, Chicago, IL, USA), rabbit anti-ITIH4 IgG (1:1000 dilution) (Bioss, Shanghai, China) and rabbit anti-MBL2 IgG (1:1000 dilution) (Aviva systems biology, San Diego, USA). Membranes were washed three times in a solution of 20 mM Tris-HCl (pH 7.5), 150 mM NaCl, and 0.05% Tween 20 and probed with horseradish peroxidase (HRP)-conjugated goat anti-mouse or anti-rabbit IgG (1:5000 dilution) (Southern Biotech, Birmingham, AL, USA) for 2 h. The reaction was developed using a chemiluminescent substrate and visualized on a chemiluminescence and fluorescence DNR Bio-imaging System (DNR, Jerusalem, Israel). Protein expression levels based on band grayscale were calculated using ImageJ^®^ software and compared.

### Statistical analyses

The data were statistically analyzed using SPSS software, version 17.0 (IBM, Armonk, NY, USA) or the programs built into the software used. Briefly, the two-tailed unpaired *t*-test was used for one comparison between two groups, and one-way analysis of variance (ANOVA) was used for comparisons among three or more groups. The receiver operating characteristic curve (ROC) was generated to evaluate the diagnostic value of the mRNA and miRNAs. The areas under the curve (AUC) with 95% confidence interval (CI) were calculated to determine the specificity and sensitivity using the statistical tests built into Prism Graph software, version 5.01 (GraphPad Software, La Jolla, CA, USA). Binary logistic regression analyses were performed using SPSS software. A default *p* < 0.05 was considered statistically significant and marked with one asterisk (*) in figures; *p* < 0.01 was considered very significantly different and marked with a double asterisk (**) in figures.

## Electronic supplementary material

Supplementary Material
