# Supplementary material for: Identification of potential urine proteins and microRNA biomarkers for the diagnosis of pulmonary tuberculosis patients
Source: Emerg Microbes Infect. 2018 Apr 11;7:63. doi: 10.1038/s41426-018-0066-5 (PMC5893550; doi:10.1038/s41426-018-0066-5)
Supplement: Supplementary file 1 — Supplementary Material [file 41426_2018_66_MOESM1_ESM.doc]

**Supplementary Material**

**Table S1 Sensitivity and specificity of biomarkers to differentiate P**TB patients from healthy persons

| **Biomarkers** | **Sensitivity** | **95% CI** | **Specificity** | **95% CI** | **AUC** |
| --- | --- | --- | --- | --- | --- |
| ITIH4-35k /MBL2/ miR-625-3p | 85.87% | 77.05%–92.26% | 87.50% | 74.75%–95.27% | 0.9260 |
| miR-625-3p | 83.70% | 74.54% -90.58% | 82.61% | 61.22% -95.05% | 0.8599 |
| MBL2 | 76.32% | 65.18% –85.32% | 78.95% | 62.68% –90.45% | 0.8251 |
| ITIH4-35k | 72.50% | 61.38% -81.90% | 60.42% | 45.27%- 74.23% | 0.7242 |
| miR-155 | 60.47% | 44.41% -75.02% | 57.14% | 34.02%- 78.18% | 0.6656 |
| RBP4 | 51.28% | 39.69% -62.77% | 62.22% | 46.54% -76.23% | 0.6168 |

Abbreviations: CI represents confidence interval; AUC represents the areas under the curve in the ROC.

**Table S2 Sensitivity and specificity of miR-625-3p in P**TB diagnosis

| **Group** | **Sensitivity** | **95% CI** | **Specificity** | | **95% CI** | **AUC** | |
| --- | --- | --- | --- | --- | --- | --- | --- |
| miR-625-3p | | | | | | | |
| HC/PTB^a^ | 83.70% | 74.54% -90.58% | | 82.61% | 61.22% -95.05% | | 0.8599 |
| HC/Sp-PTB^b^ | 97.50% | 86.84% - 99.94% | | 82.61% | 61.22%- 95.05% | | 0.9402 |
| HC/Sn-PTB^c^ | 70.00% | 50. 60%-85.27% | | 69.57% | 47.08%-86.79% | | 0.7109 |

Abbreviations: a means the comparison between health and PTB groups; b means the comparison between health and Sp-PTB groups; c means the comparison between health and Sn-PTB groups; d means the comparison between Sn-PTB and Sp-PTB groups.

**Table S3** List of combined diagnostic models

| Combined diagnostic models | Area | Std. Error | Asymptotic 95%  Confidence Interval | |
| --- | --- | --- | --- | --- |
|  |  |  | Lower Bound | Upper Bound |
| ITIH4+MBL2+miR-625 | 0.926 | 0.022 | 0.883 | 0.969 |
| ITIH4+MBL2+miR-625+RBP4 | 0.905 | 0.022 | 0.890 | 0.978 |
| ITIH4+MBL2+miR-625+miR-155 | 0.914 | 0.027 | 0.852 | 0.958 |
| ITIH4+MBL2+miR-625+RBP4+miR-155 | 0.911 | 0.027 | 0.858 | 0.964 |

**Table S**4 Primers used in this study for qRT-PCR

| **Genes** | **Direction** | **Sequences (5’-3’)** |
| --- | --- | --- |
| ITIH4-35k | Sense | AGTCACCAAACCCGATGACC |
|  | Antisense | GCTCCAGCTGAGTGGACATT |
| RBP4 | Sense | TCCAGAAAGGAAATGATGAC |
|  | Antisense | AAAACACGAAGGAGTAGCTG |
| MBL2 | Sense | GCTAGGCTGCTGAGGTTTCTT |
|  | Antisense | GGGCTGGCAAGACAACTATTAG |
| U6 | Sense | CTCGTTCGGCAGCACA |
|  | Antisense | AACGCTTCACGAATTTGCGT |
| miRNA-625-3p | Sense | GACTATAGAACTTTCCCCCTCA |
| miRNA-155-5P | Sense | TTAATGCTAATCGTGATAGGGGT |
